# Monitoring ovarian cycles, pregnancy and post-partum in captive marsh deer (*Blastocerus dichotomus*) by measuring fecal steroids

**DOI:** 10.1093/conphys/cox073

**Published:** 2018-01-25

**Authors:** Bruna Furlan Polegato, Eveline dos Santos Zanetti, José Maurício Barbanti Duarte

**Affiliations:** Deer Research and Conservation Center (NUPECCE—Núcleo de Pesquisa e Conservação de Cervídeos), Departamento de Zootecnia, Faculdade de Ciências Agrárias e Veterinárias, Universidade Estadual Paulista, Via de Acesso Professor Paulo Donato Castellane s/n, Jaboticabal, SP 14884-900, Brazil

**Keywords:** Neotropical deer, fecal estrogens, fecal progestins, enzyme immunoassay, cloprostenol

## Abstract

The marsh deer is an endangered species from the marshlands of central South America. This study aimed to characterize certain aspects of the reproductive physiology of marsh deer hinds, including the duration and fecal progestins profile of the estrous cycle, pregnancy and post-partum periods, and evaluate the effect of cloprostenol administration on this species. The experimental group consisted of six females and one fertile male marsh deer. During monitoring of the estrous cycle, the fresh fecal samples were collected daily and, during pregnancy, they were collected twice weekly. The hormonal profile obtained from daily fecal samples indicated that the mean duration of the estrous cycle was 21.3 ± 1.3 days (6.4 days inter-luteal phase and 14.8 days luteal phase; *n* = 16 estrous cycles). The mean concentration of fecal progestins in the inter-luteal phase was 834 ± 311 ng g^−1^, in the luteal phase was 3979 ± 1611 ng g^−1^, value between them was 1457 ng g^−1^. No significant difference in fecal estrogen concentrations was determined during the estrous cycle. The corpora luteum was not responsive to cloprostenol until Day 6 of the estrous cycle, the period previously described as the inter-luteal phase. Half the females became pregnant following treatment with cloprostenol and two others were fertilized in their natural estrous cycle. Four females delivered fawns, and the mean duration of pregnancy was 253 ± 4 days. Fecal progestin concentrations were similar to those of the estrous cycle during the first 11 weeks of pregnancy and increased significantly ( > 15250 ng g^−1^) thereafter, providing a presumptive diagnosis guideline. Within 60 days of post-partum analyses, 75% of the deer exhibited behavioural estrus and/or ovarian activity. This study generated a broader understanding of the marsh deer species concerning the production of consistent data related to its reproduction. This knowledge can be used to assist the reproductive management of this species and, consequently, to promote its conservation.

## Teaser

Lay summary: This study aimed to characterize some aspects of the reproductive physiology of female marsh deer, such as length and fecal progestins profile of estrous cycle, pregnancy and post parturition periods, information that we consider an important and decisive factor for the elaboration of conservation actions of Neotropical deer species.

## Introduction

The marsh deer (*Blastocerus dichotomus*) is the largest Neotropical deer and shows high specificity for humid environments (Duarte, 1996; Pinder, 1996; Piovezan *et al.*, 2010). Its original geographical distribution has been drastically reduced due to the expansion of human activity and ~65% of the areas once occupied by the species have been lost over the past 40 years (Weber and González, 2003; Márquez *et al.*, 2006). Currently, the marsh deer is either threatened with extinction or is extinct in certain areas and is classified as vulnerable (Duarte *et al.*, 2016). It is also categorized as Appendix I according to the Convention on International Trade in Endangered Species of Wild Fauna and Flora (CITES), primarily due to habitat loss.

Knowledge regarding this species is scarce and lacunae exist, particularly concerning its reproduction, making it more susceptible in cases of disaster or imminent threats. Thus, the adoption of more intensive conservation measures is recommended.

In 1998, a program was created to conserve this species in captivity, aimed at maintaining a genetic stock for use in future reintroductions (Figueira *et al.*, 2005). The success of the program depends on management to ensure gene flow between the institutions involved and maximization of the genetic diversity of the population (Zanetti and Duarte, 2008; Duarte, 2010; Piovezan *et al.*, 2010). It is known that in small populations, the loss of genetic variability is mainly due to genetic drift and inbreeding and that the use of assisted reproduction techniques can potentialize reproduction, guaranteeing the formation of updated genomic banks and facilitating reproductive management, thus avoiding such losses (Wildt *et al.*, 1997; Wildt and Wemmer, 1999).

The use of assisted reproduction techniques, however, depends on understanding certain features of the species’ reproductive biology, such as the onset of puberty, reproductive seasonality, ovarian cyclicity, luteal function and pregnancy (Duarte and Garcia, 1995). Establishing efficient methods to manipulate the estrous cycle, including techniques for estrus synchronization, are fundamental to achieve artificial insemination and embryo transfer (Pickard *et al.*, 2001), which have great potential to assist conservation programs. The establishment of reliable methods to access reproductive events permits not only the use of reproductive biotechniques, but also monitoring and reproductive management of captive and wild populations (Wasser *et al.*, 1995; Borjesson *et al.*, 1996).

However, to date, knowledge concerning the reproductive biology of this species is scarce and manipulation of the estrous cycle in the marsh deer has yet to be described. Improving current understanding of the reproductive physiology of the species can be partly ascertained by characterizing the profile of reproductive steroid hormones, which is an alternative method of monitoring the phases of the reproductive cycle. However, the use of traditional methods based on containment and periodic blood samples to measure the concentrations of these hormones may be inadequate, since these deer present strong resistance to handling and sensitivity to stress (Nunes and Duarte, 2010; Pereira and Polegato, 2010). When subjected to such conditions, interrupted or failed reproduction, catabolic damage to the deer and increased risk of developing trauma and other injuries can occur (Lasley *et al.*, 1989; Monfort *et al.*, 1990; Morrow and Monfort, 1998; Hamasaki *et al.*, 2001; Monfort, 2002; Schoenecker *et al.*, 2004).

The use of non-invasive methods to monitor reproductive activity in wild species enables studies to be developed on such species. Measuring the metabolites of steroid hormones that are excreted in the feces, urine and saliva is an effective method and the ease afforded by the use of feces make it a safe and practical alternative (Pereira and Polegato, 2010). Thus, the objectives of this study were (i) to validate the measurement of reproductive steroid hormones and their fecal metabolites for reproductive monitoring in the species *Blastocerus dichotomus*, (ii) characterize certain parameters related to the reproductive physiology of the female, (iii) provide adequate methods to differentiate the phases of the reproductive cycle and (iv) evaluate the effect of cloprostenol (a synthetic analogue of prostaglandin F_2α_) administration in this species. These measures aim to provide a better understanding of the physiology and dynamics of the corpus luteum (CL) of the species.

## Methods

### Animals

The experimental group consisted of six adult females and one fertile male marsh deer (Table 1). The group was maintained at the installations of the Deer Research and Conservation Center (NUPECCE) of São Paulo State University (UNESP), Jaboticabal, SP, Brazil. At the onset of the study, the females were isolated from the male for 6 months, ensuring that they were not pregnant during monitoring of the estrous cycle. The deer were also submitted to management conditioning for three months. Management of the diet and feeding were standardized with food offered in individual stalls between 5 and 6 pm. This comprised ~1 kg of concentrated feed (equine feed: Omolene®, Purina Co., Paulinia, SP, Brazil), 2 kg of forage (*Glicyne max*, *Morus Alba* or *Neonotonia wightii*) and water *ad libitum*.

**Table 1:** Characteristics of the seven deer of the species *Blastocerus dichotomus* that composed the experimental group

Deer	Age (years)	Weight (kg)	Procedence	Reproductive history	Behaviour
F36	8 (~03/1998)	74.9	Wild^b^	Pluriparous	Reactive^c^
F105	8 (~05/1998)	81.2	Wild	Pluriparous	Reactive
F261	6 (16/05/2000)	89.3	Captive	Pluriparous	Reactive
F262^a^	5 (15/03/2001)	87.0	Captive	Pluriparous	Non-reactive^d^
F269	3 (03/03/2003)	63.1	Captive	Nuliparous	Very reactive^e^
F270	3 (15/10/2003)	62.5	Captive	Nuliparous	Non-reactive
M52	12 (~12/1995)	~110	Wild	–	Reactive

F = female; M = male. ^a^Female in lactation. ^b^Captured in the Rio Paraná basin. ^c^Deer showed strong resistance to being touched (except when in estrus), but tolerated the management procedures well. ^d^Deer tolerated being touched. ^e^Deer showed strong resistance to being touched and only tolerated the management procedures with restrictions.

### Monitoring the estrous cycle

During the first 3 months, the females were transferred from individual stalls (4 × 4 m^2^) to a paddock (30 × 50 m^2^) every day (between 7 and 9 am), where they remained together. In the afternoon (between 5 and 6 pm), the deer were led back to the stalls, where food was offered. In these periods of handling, the females were observed closely to identify signs of behavioural estrus. These were characterized by the female allowing approximation (even among deer presenting reactive behaviour), evidence of a stop reflex when dorsal pressure was exerted by the examiner, abundant mucoid vulvar discharge and vulvar hyperemia.

### Manipulation of the estrous cycle and female conception

To improve the current understanding concerning the estrous cycle in this species, the females were submitted to two treatments to synchronize estrous cycle (*n* = 3 deer per treatment) consisting of two applications of a synthetic analogue of prostaglandin F_2α_, cloprostenol sodium (2 mL, 530 ug i.m.—Ciosin®, Schering Plough Coopers®, Brazil) (Fisher *et al.*, 1994; Asher *et al.*, 1995) at different intervals. The applications were performed with a 12-day interval in treatment 1 and a 6-day interval in treatment 2 (Asher *et al.*, 1995). The day of the estrous cycle at the time of treatment was determined for each female. Following the second application of the drug, the females were placed in contact with a fertile male, twice daily (8 am and 5 pm), and copulation was allowed. The females that did not show apparent estrus following the treatment received an additional dose of cloprostenol (2 mL, 530 μg i.m.) ~15 days after the initial dose, following the same management scheme adopted previously. Females who redisplayed behavioural estrus following the synchronization treatment and copulation were placed in contact with the male again and at least two copulations were observed.

### Monitoring pregnancy and the post-partum period

The females were maintained in the paddock during pregnancy and were brought back to the stalls on the day preceding the collection of fecal samples. About 15 days prior to parturition and during the post-partum period (60 days), the females were maintained exclusively in the stalls to facilitate greater control of the parturition, the handling of fawns and to permit monitoring of post-partum estrus. During this period, the deer were observed closely (at 8 am and 6 pm) to identify signs of behavioural estrus, as described before.

### Collection and processing of fecal samples

Fresh fecal samples were always collected in the morning, between 8 and 10 am. During monitoring and manipulation of the estrous cycle the samples were collected daily; during pregnancy (between copulation and parturition), they were collected twice weekly, on Tuesdays and Fridays; and in the post-partum period, on alternate days. Following collection, the samples were stored in plastic bags, identified and frozen at −20°C. The method described by Graham *et al.* (2001) was used to extract the metabolites (estrogen and progestin) from the fecal samples. Briefly, 5 ml of 80% methanol was added to 0.5 g of lyophilized and triturated sample material. The mixture was vortexed for 30s, agitated for 12 h in a horizontal homogenizer, and vortexed again for 15 s. Following centrifugation at 1500*g* for 20 min, the supernatant was separated and constituted the final extract.

### Determining hormone concentrations

The concentrations of progestins and estrogens in fecal extracts from the estrous cycle were analysed by enzyme immunoassay (EIA). Only the concentration of fecal progestins was analysed in fecal extracts collected during pregnancy and the post-partum period. The antibodies CL425 and R4972 (University of California, Davis, CA, USA) were used for fecal progestins (P) and fecal estrogens (E2), respectively. These antibodies were chosen because they present high cross-reactivity with metabolites excreted in the feces of *B. dichotomus*, namely, 5α- and 5β-pregnanes and 17β-estradiol (Polegato, 2004). All the fecal extracts were diluted in dilution buffer at 1:500 (estrous cycle and early pregnancy), 1:1500 (mid-pregnancy) and 1:2500 (late pregnancy) for P and 1:32 (estrous cycle) for E2. The samples were assayed in duplicate. The validation of hormone concentrations was performed as described by Brown *et al.* (2004): (i) by the significant recovery of properly diluted exogenous hormones added to fecal samples (*y* = 1.149*x* – 2.2556, *r*^2^ = 0.99 and *y* = 1.088*x*+1.6021, *r*^2^ = 0.99 for P and E2, respectively); (ii) by comparing a curve parallel to the standard curve formed by the pool of fecal extracts prepared by serial dilution in dilution buffer (*R*^2^ = 0.9914 and *R*^2^ = 0.9744, respectively, for fecal P and E2; and (iii) due to the physiological relevance of the results obtained when different phases of the reproductive cycle were compared. The intra-assay coefficients of variation were < 10% for all the hormones and controls evaluated. The interassay coefficients of variation were 10.1% (~35% binding, *n* = 57 plates) and 12.1% (~75% binding, *n* = 57 plates) for P and 7.4% (~20% binding, *n* = 29 plates) and 13.9% (~50% binding, *n* = 29 plates) for E2. Assay sensitivity was 0.78 ng g^−1^ (93.7% binding) for P and 1.95 ng g^−1^ (89.5% binding) for E2. All fecal data are expressed on a dry-weight basis.

### Statistical analysis

Data analyses of the estrous cycle was performed based on the model proposed by Thompson *et al.* (1998), with modifications. The three lowest values of progestin concentration of each estrous cycle were considered basal and from these, the mean and standard deviation (SD) were calculated. Values greater than the limit (mean + 2SD) were considered indicative of the luteal phase and values below this were considered indicative of the inter-luteal phase. To calculate the duration of the estrous cycle, the day the concentration of fecal progestins reached the value indicative of the inter-luteal phase was considered the day one (D1) of the cycle. To determine the minimum concentration of fecal progestins indicative of pregnancy, the mean of the first month that showed significantly different concentrations of fecal progestins from the estrous cycle was subtracted from the standard error of the mean (SEM).

The data are presented as the mean ± SEM and comparisons between the deer, the estrous cycle phases (luteal and inter-luteal) and the different months of pregnancy and anestrus were performed using repeated-measures analysis of variance (ANOVA), followed by the Scott-Knott test. The fecal hormone concentration values were submitted to analysis of variance following logarithmic transformation of the hormone data (Morrow *et al.*, 1995). Correlation between the variables was determined by Pearson’s correlation test.

The E2:P ratio was calculated for the days on which both hormones were analysed. All the analyses were performed using the SAS software (SAS Institute Inc., Cary, NC, USA) and the significance level for all statistical tests was 5% (*P* < 0.05).

## Results

### Estrous cycle

**Figure 1:**
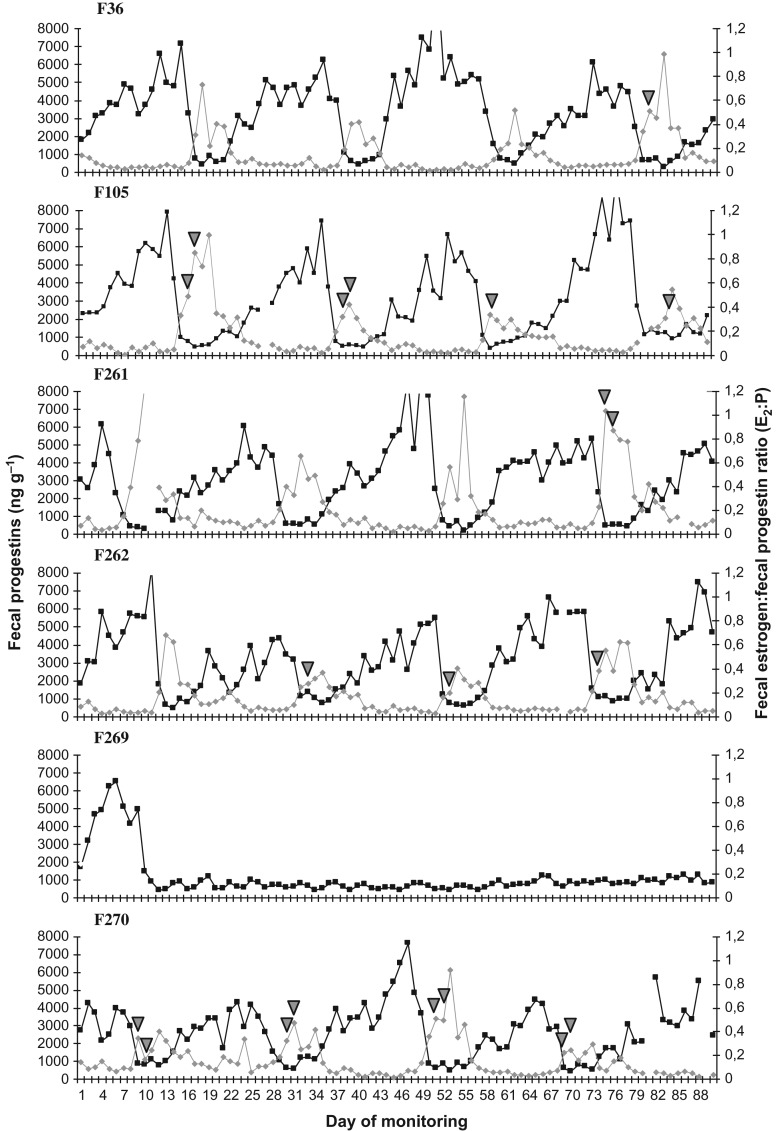
Monitoring the estrous cycle by determining the concentrations of fecal progestins (black line) and fecal estrogen:fecal progestin, ratio (gray line) in six females of *Blastocerus dichotomus*. The arrows indicate the day behavioural estrus was detected.

A total of 16 complete estrus cycles were evaluated, with a mean duration of 21.3 ± 1.3 days (range: 19–23 days), as determined by the hormonal profiles. The mean duration of the inter-luteal phase of the cycle was 6.4 ± 1.2 days, while the mean duration of the luteal phase was 14.8 ± 1.3 days. These means include data from five of the six females, since F269 remained anestrous from Day 12 of monitoring. No differences (*P* > 0.05) were observed in estrous cycle duration among females or different cycles of the same female (Fig. 1 and Table 2). Of the 20 estrous periods observed using the fecal progestin profile, 13 (65%) were also detected by behaviour. Regular detection of behavioural estrus was only possible in two females (F105 and F270; *n* = 8 estrous periods) (Fig. 1), and in all cases, these behaviours were correlated with hormonal profiles (Fig. 1). The duration of estrus behavioural ranged from 1 to 2 days (*n* = 13 estrous periods).

**Table 2:** Characteristics of the estrous cycle and pregnancy in five female *Blastocerus dichotomus*

	Estrous cycle						Pregnancy										
Deer	Observed cycles	Inter-lutel phase (days)	Luteal phase (days)	Length (days)	[P] Inter-luteal (ng g^−1^)	Luteal progestins (ng g^−1^)	Pregnancy length (days)	Pregnancy Weeks	M1 (ng g^−1^)	M2 (ng g^−1^)	M3 (ng g^−1^)	M4 (ng g^−1^)	M5 (ng g^−1^)	M6 (ng g^−1^)	M7 (ng g^−1^)	M8 (ng g^−1^)	M9 (ng g^−1^)
F36	1st	5	16	21	663	4034	249 (F) 4.1 kg***	W1	2607	5013	4226	17 850	25 356	31 209	23 225	25 946	22 524
	2nd	6	16	22	733	5311		W2	6511	8730	6289	11 870	24 109	22 724	29 207	35 295	18 535
	3rd	4	16	20	734	3384		W3	7324	5469	6830	16 038	27 175	14 431	24 992	28 463	–
	4th	–	–	–	–	–		W4	6013	5352	10 841	19 015	30 120	16 899	26 533	21 445	–
	Mean	5 a	16 a	21 a	710 a	4243 a		Mean	5659 a	6127 a	7469 a	15 791 a	27 072 a	21 316 a	25 990 a	27 368 a	21 195 a
F105*	1st	9	13	22	896	4052	–	W1	–	–	–	–	–	–	–	–	–
	2nd	8	13	21	757	3945		W2	–	–	–	–	–	–	–	–	–
	3rd	7	16	23	821	4815		W3	–	–	–	–	–	–	–	–	–
	4th	–	–	–	–	–		W4	–	–	–	–	–	–	–	–	–
	Mean	8 a	14 a	22 a	824 a	4270 a		Mean	–	–	–	–	–	–	–	–	–
F261**	1st	8	15	23	773	3448	257 (M) 4.5 kg***	W1	1162	8409	7422	14 517	24 562	21 282	32 233	42 854	13 816
	2nd	6	16	22	682	4519		W2	6471	4847	7837	18 144	22 119	34 923	36 984	33 514	22 860
	3rd	7	16	23	671	3919		W3	6100	6477	9592	18 111	25 027	32 952	38 844	29 361	29 787
	4th	–	–	–	–	–		W4	8479	6495	12 383	19 284	26 127	28 192	39 293	16 243	–
	Mean	7 a	15.7 a	22.7 a	708 a	3962 a		Mean	5407 a	6569 a	9651 a	17 515 a	24 645 a	29 338 a	37 112 bc	32 267 a	20 628 a
F262	1st	5	14	19	851	2869	250 (F) 4.9 kg***	W1	1737	4164	6025	18 398	14 421	34 540	36 790	58 183	64 994
	2nd	5	15	20	1035	3364		W2	5925	6697	–	18 509	13 393	26 369	36 243	61 606	48 053
	3rd	7	15	22	925	4496		W3	5718	6465	8469	13 266	31 104	38 489	35 844	56 000	–
	4th	–	–	–	–	–		W4	6706	4341	9292	10 889	28 000	31 909	53 485	59 944	–
	Mean	5.7 a	14.7 a	20.3 a	937 a	3576 a		Mean	4784 a	5565 a	7929 a	15 266 a	22 427 a	32 726 a	42 024 b	58 934 b	59 347 b
F270**	1st	5	15	20	907	2913	257 (M) 2.4 kg***	W1	2265	8370	6206	17 252	18 479	24 363	29 552	42 914	23 525
	2nd	6	15	21	973	4108		W2	5454	6998	9125	20 450	19 573	30 777	26 953	35 536	27 449
	3rd	7	12	19	770	2850		W3	5760	10 264	6440	22 605	15 210	44 676	25 312	23 976	24 484
	4th	6	16	22	718	3007		W4	6310	7244	8317	23 124	23 876	34 467	33 115	29 484	–
	Mean	6 a	14.5 a	20.5 a	842 a	3219 a		Mean	4650 a	8220 a	7562 a	20 862 a	19 796 a	33 671 a	29 221 ac	32 978 a	25 287 a
Mean ± EPM		6.4 ± 1.2	14.8 ± 1.3	21.3 ± 1.3	834 ± 311A	3979 ± 1611 B	253 ± 4		5123 ± 1224 B	6657 ± 1035 B	8212 ± 1232 B	17 409 ± 2167 C	23 484 ± 3547 C	29 494 ± 4670 D	33 803 ± 4625 D	38 068 ± 7661 D	29 450 ± 8100 D

*Female had a miscarriage in early pregnancy.

**Females that were ill during the final trimester of pregnancy.

***Sex (male [M] or female [F]) and birth weight of the fawn.

Means within column with uncommon and lowercase letters (a, b and c) differ (*P* < 0.05) by the Scott-Knott test; means within row with uncommon and capital letters (A, B, C and D) differ (*P* < 0.05) by the Scott-Knott test.

The mean concentration of fecal progestins for the inter-luteal phase of the estrous cycle was 834 ± 311 ng g^−1^ (range: 393–1431 ng g^−1^) and differ (*P* < 0.05) from the luteal phase (3979 ± 1611 ng g^−1^ (range: 1498–11 364 ng g^−1^)), as determined by the individual profiles (Table 2). Based on the criteria defined above, a concentration of 1457 ng g^−1^ constitutes the limit value between the two phases. However, it was not possible to differentiate deer in the inter-luteal phase from anestrus deer. No significant differences in E2 concentrations were verified during the estrous cycle (*P* > 0.05); however, differences (*P* < 0.05) in the ratio of the concentrations of this hormone (E2) and P concentrations (E2:P) were observed. A negative correlation was determined between the concentration of P and the E2:P ratio (*r *= −0.39, *P* < 0.001). The peak values obtained for the E2:P ratio coincided with behavioural estrus or occurred one day after behavioural estrus (Fig. 1).

### Manipulation of the estrous cycle

**Figure 2:**
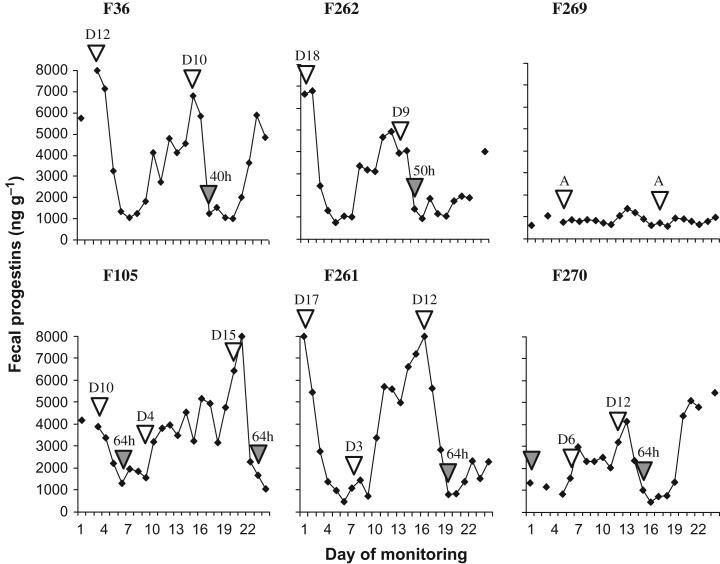
Excretion of fecal steroid hormone metabolites of six female *Blastocerus dichotomus* during two different estrus synchronization treatments using cloprostenol (F36, F262, F269: two applications of cloprostenol at 12-day intervals; and F105, F261, F270: two applications of cloprostenol at 6-day intervals). White arrows indicate the day in the estrous cycle which each female was on the day of treatment administration (A = anestrus) and gray arrows indicate the day behavioural estrus was detected (time, in hours, between treatment administration and detection of behavioural estrus).

When used up to Day 6 of the estrous cycle, cloprostenol did not trigger an effective luteolytic response. All the females who responded to the drug exhibited behavioural estrus, which began on average 58 h following administration (range: 40–64 h). One deer (F269) was in anestrus and did not respond to the treatment (Fig. 2).

### Pregnancy

Three females became pregnant following treatment with cloprostenol (F105, F262 and F270) and two others were fertilized in subsequent natural estrous periods (F36 and F261). F105 was fertilized, but miscarried in early pregnancy according to a prolonged luteal activity for ~60 days following copulation. Of the four females in which pregnancy went to term, two (F261 and F270) became ill in the final trimester. The mean duration of pregnancy was 253 ± 4 days (*n* = 4).

Endocrine characterization of pregnancy showed that the concentrations of fecal progestins remained consistent with the values of the luteal phase of the estrous cycle until about the third month of pregnancy (*P* > 0.05), after which they began to gradually increase. In the fourth month of pregnancy, the concentration of progestins nearly doubled compared with previous months (*P* < 0.05), reaching a peak in the eighth month, at which point the mean concentrations were 6-fold greater than those obtained during the initial phases (*P* < 0.05) (Table 2). Concentrations of fecal progestins ≥15 250 ng g^−1^ were indicative of pregnancy.

### Post-partum period

The concentrations of fecal progestins began to decline in late pregnancy (vary from 1 week to 1 month before parturition) and only achieved basal concentrations following parturition (4–8 days). During analysis of the post-partum period, three (F261, F262 and F270) of the four deer studied presented a cyclic pattern in the excretion of fecal progestins, which demonstrates the resumption of ovarian activity and confirms the existence of post-partum estrus in this species. A common characteristic among these deer was the occurrence of a short cycle, with lower concentrations of fecal progestins preceding normal cycles (Table 3).

**Table 3:** Characteristics of the post-partum period in four female *Blastocerus dichotomus*

Deer	[P] Parturition (ng g^−1^)	[P] Basal^a^ (days)	Anestrus^b^ (days)	First luteal phase		Second luteal phase	
				Duration (days)	Mean [P] (ng g ^−1^)	Duration (days)	Mean [P] (ng g ^−1^)
F36	16 553	7	Over 53	–	–	–	–
F261^c^	12 412	5	14	4	2052	12	4640
F262	21 230^e^	10^f^	6	4	1963	14	2592^g^
F270^c,d^	9907	3	14	6	2314^g^	12	4591^g^

[P] Fecal progestin concentration.

^a^Period for [P] to achieve inter-luteal phase concentration.

^b^Considering an inter-luteal phase of 6 days.

^c^Illness during the final trimester of pregnancy.

^d^Lost fawn; deer did not lactate.

^e^[P] 5 days post-partum.

^f^Behavioural estrus 6 days post-partum.

^g^Behavioural estrus before [P] achieved luteal phase concentration.

## Discussion

Hormonal analyses performed on fecal samples proved to be an efficient alternative for monitoring reproductive events in the marsh deer. The results obtained in this study showed that this technique has the potential to clarify reproductive status in this species, since it characterizes and differentiates the different phases of the reproductive cycle. Given its non-invasive nature, it can be applied when monitoring captive and wild populations, even when dealing with species that are highly sensitive to stress (Nunes and Duarte, 2010).

The mean duration of the estrous cycle obtained in this study was slightly shorter than that previously observed for this species (24 days; Duarte and Garcia, 1997; Schwarzenberger and Dreben, 1998), and comparable to other cervid species with similar body size (17–21 days for *Axis axis* (Chapple *et al.*, 1993), 13–22 days for *Cervus unicolour* and 15–24 days for *Cervus elaphus* (Asher *et al.*, 1997), 14–23 days for *Cervus eldi thamin* (Monfort *et al.*, 1990), 17–21 days for *Cervus nippon taiouanus* (Liu *et al.*, 2002)). The difference between the luteal and inter-luteal phases of the estrous cycle was evident when monitoring fecal progestins; however, no difference was verified in fecal estrogen concentrations. It is possible that the failure to detect fecal estrogen peaks could be due to lower concentrations of circulating estrogen or because it is excreted as a urinary metabolite, as reported for other ungulate species (Schwarzenberger *et al.*, 1996). Research indicates that estrogens can induce adult female sheep to express behavioural estrus and that progesterone enhances the role of estrogen in sexual behaviour (Keverne *et al.*, 1983). Thus, the E2:P ratio was more effective at indicating the time of ovulation than isolated analysis of steroid hormones, as suggested previously for *Gazella dama mhorr* (Pickard *et al.*, 2001), for *Mazama gouazoubira* (Zanetti *et al.*, 2010) and as described in humans (Lenton *et al.*, 1989). The peak values of the E2:P ratio coincided with the period of behavioural estrus, such that the behavioural data assured the accuracy of endocrine monitoring.

Cloprostenol adequately promoted luteolysis in cyclic female marsh deer, suggesting that it could be an important drug for manipulating the estrous cycle of this species. However, the action of this drug is directly related to the presence of a functional CL (Asher *et al.*, 1993; Whitley and Jackson, 2004). Cloprostenol was unable to promote luteolysis in the marsh deer when treatment was administered while the deer was in anestrus or when applied up to Day 6 of the estrous cycle, during which the CL is absent or hypofunctional. This finding is similar to that observed for *C. elaphus* (Asher *et al.*, 1995) and for most mares (Pinto, 2013), which proved to be insensitive to the action of prostaglandin F_2α_ up to Day 6 of the estrous cycle and contrasts with that observed for sheep (Rubianes *et al.*, 2003) and some mares (Pinto, 2013), in which luteolysis can be induced from day three of the estrous cycle. This refractory period of the CL coincides with the duration of the inter-luteal phase of the estrous cycle of the species *B. dichotomus*, in which the CL is still in formation, secreting small quantities of progesterone and basically consists of small luteal cells that are unresponsive to prostaglandin F_2α_ (Berisha and Schams, 2005).

The time until the onset of behavioural estrus following treatment with cloprostenol was similar to that previously reported for other deer species, such as *M. gouazoubira* (40–69 h) (Zanetti *et al.*, 2010) and *Dama dama* (42–64 h) (Jabbour *et al.*, 1993) and this variation is related to the follicular stage present at the moment of luteolysis induction (Rubianes *et al.*, 2003; Barros and Ereno, 2004).

All the deer that responded to cloprostenol, i.e. displaying behavioural estrus, had ovulation and formation of the CL, as determined by the concentrations of fecal progestins. The pregnancy rate following synchronization with this drug was 50% (3/6); however, although this is low, it is numerically superior than that reported for *Orix dammah* (37.5% (Morrow *et al.*, 2000)) and *D. dama* (40.7% (Jabbour *et al.*, 1993)) using similar treatment protocols, followed by artificial insemination. In this case, the different forms of breeding could have influenced the difference between the studies, as well as the small number of deer that constituted the experimental group herein.

The mean gestation period observed for this species was shorter than that described previously (271 days) (Frädrich, 1995) and is compatible with other uniparous cervid species of similar body size (*C. elaphus* (Asher *et al.*, 2005), *C. elaphus nannodes* (Stoops *et al.*, 1999), *C. eldi thamin* (Monfort *et al.*, 1990), *C. nippon* (Hamasaki *et al.*, 2001), *D. dama* (Willard *et al.*, 1998), *Rangifer tarandus tarandus* (Ropstad *et al.*, 2005)). However, the gestation period can be extended if the female suffers severe food restriction and, in some cases, can lead to the birth of fawns with body mass index below normal (Verme, 1965). Due to the correlation between fawn mortality and its body weight at birth, females can significantly ravage their energy reserves to try to ensure that the fawn is born with an adequate body mass (García *et al.*, 2006). This plasticity in the physiological response was observed in the two females that became ill in the final trimester of pregnancy and in which the period of gestation was extended by about a week, apparently as a form of compensation. However, despite presenting a severely diminished body mass and prolonged period of gestation, F270 produced a fawn with low birth weight, which died one day following parturition.

The pattern of fecal progestin excretion during pregnancy was similar for all the deer in the study. Based on the hormonal data, a presumptive diagnosis of pregnancy in *B. dichotomus* can be determined from the second trimester onward (from the four month of pregnancy). This finding is similar to that reported for other cervid species (*Capreolus capreolus* (Sempéré, 1977), *C. elaphus nelsoni* (White *et al.*, 1995; Garrott *et al.*, 1998), *C. elaphus nannodes* (Stoops *et al.*, 1999), *C. eldi thamin* (Monfort *et al.*, 1990), *D. dama* (Willard *et al.*, 1998)) and is due to the fact that the placenta synthesizes progesterone in most deer species during pregnancy. Steroidogenesis is obviously faster in the luteal tissue than in the placenta, thus during early pregnancy, when the placental volume/area is small, the importance of this source of progesterone synthesis and secretion is limited. During mid-pregnancy, the placental volume becomes much greater than the luteal volume and it is likely that the placenta is a physiologically significant source of steroid synthesis from this period onward (Flood *et al.*, 2005).

Post-partum estrus, which has previously been described in this species (Frädrich, 1995), was observed in three of the four deer in which pregnancy went to term and seems to be related to the abundance of food and habitat stability (Robbins, 1983). This is probably due to the association of a marked decline in progestogen concentrations and a sharp increase in estrogen concentrations observed in some ungulates during parturition. This provides a favourable hormonal environment for the expression of behavioural estrus (Pereira *et al.*, 2006). The resumption of ovarian activity was characterized by the hormonal profile and presented some peculiarities, such as the appearance of a shorter cycle showing a lower concentration of fecal progestin excretion preceding the normal estrous cycles. This cycle could be related to the luteolytic influence of the involuting uterus due to an increased and prolonged release of prostaglandin F_2α_ and an incomplete restoration of LH release leading to insufficient follicular growth and maturation, as previously reported in sheep (Schirar *et al.*, 1989). Thus, as documented in the Mohor gazelle (Pickard *et al.*, 2001), the conception rate in post-partum estrus may be lower than normal, suggesting failure of the reproductive tract, which may be unable to sustain pregnancy (Pereira *et al.*, 2006).

## Conclusion

This study validated the measurement of reproductive steroid hormones and their fecal metabolites for reproductive monitoring in the species *B. dichotomus*, provided adequate methods to differentiate the phases of the reproductive cycle and evaluated the effect of cloprostenol (a synthetic analogue of prostaglandin F_2α_) administration in this species; generating a broader understanding of the marsh deer species concerning the production of consistent data related to its reproduction. This knowledge can be used to assist the reproductive management of this species and, consequently, to promote its conservation.
